# Videokymographic index of glottic function: an analysis of diagnostic accuracy

**DOI:** 10.1590/2317-1782/20212021214en

**Published:** 2022-10-17

**Authors:** Alice Braga de Deus, Roberto da Costa Quinino, Marco Aurélio Rocha Santos, Ana Cristina Côrtes Gama

**Affiliations:** 1 Programa de Pós-graduação em Ciências Fonoaudiológicas, Faculdade de Medicina, Universidade Federal de Minas Gerais – UFMG - Belo Horizonte (MG), Brasil.; 2 Departamento de Estatística, Instituto de Ciências Exatas, Universidade Federal de Minas Gerais – UFMG - Belo Horizonte (MG), Brasil.; 3 Hospital das Clínicas, Universidade Federal de Minas Gerais – UFMG - Belo Horizonte (MG), Brasil.; 4 Departamento de Fonoaudiologia, Faculdade de Medicina, Universidade Federal de Minas Gerais – UFMG - Belo Horizonte (MG), Brasil.

**Keywords:** Kymography, Voice, Dysphonia, Diagnostic Test Approval, Larynx

## Abstract

**Purpose:**

To develop the Videokymographic Index of Glottic Function (VIGF), a composite indicator from digital videokymography parameters, captured by high-speed videolaryngoscopy exams of women with and without laryngeal alterations of behavioral etiology.

**Methods:**

The sample consisted of 92 women aged between 18 and 45 years. Fifty-five (55) women with behavioral dysphonia, presenting with laryngeal and voice alterations, and thirty-seven (37) women without any laryngeal and voice alterations. Voice evaluation was performed by consensus via an auditory-perceptual analysis of the sustained vowel /a/ at a habitual pitch and loudness. Voice classification was obtained by means of a general degree of dysphonia, where G0 indicated neutral voice quality and G1 to G3 indicated altered voice quality. Laryngeal images were captured via digital videokymography analysis of a sustained vowel /i/ at a habitual pitch and loudness. The VIGF was based on the midpoint of the glottal region for analysis. Logistic regression was performed using the MINITAB 19 program.

**Results:**

Logistic regression was composed of two stages: Stage 1 consisted of the analysis of all variables, where the maximum opening and closed quotient variables showed statistical significance (p-value <0.05) and the model was well adjusted according to the Hosmer-Lemeshow test (p-value=0.794). Stage 2 consisted of the re-analysis of the selected variables, also showing a well-adjusted model (p-value=0.198). The VIGF was defined as follows: VIGF=e^(8.1318-0.2941AbMax-0.0703FechGlo)/1+e^(8.1318-0.2941AbMax-0.0703FechGlo).

**Conclusion:**

The VIGF demonstrated a cut-off value equal to 0.71. The probability of success was 81.5%, sensitivity 76.4%, and specificity 89.2%.

## INTRODUCTION

Voice is a primary communication tool that transmits important speaker characteristics^(1)^. Phonation is coordinated by neurophysiological mechanisms, which require harmony and integrity of the phonation apparatus^(1)^.

Voice is the result of the integration of multiple dimensions. Hence, the classification of dysphonia can be based on several criteria. Behavioral dysphonias have an etiology related to vocal behavior^(2)^. Mucosal tissue lesions, such as vocal nodules, edema, polyps, among others, exemplify this dysphonia condition^(1)^.

Phonatory effort related to behavioral dysphonia may limit patients' quality of life^(3)^. Diagnosis is multidisciplinary and includes laryngeal exams as fundamental tools that describe vocal fold vibration patterns and glottal cycle phases^(4)^.

An otolaryngology evaluation utilizes several methods and technologies. Videolaryngostroboscopy is the method that allows visualization of the mucosal wave movement of the vocal fold vibration patterns^(4)^. High-speed videoendoscopy (HSV) is an instrument capable of capturing 2000 to 6000 images per second, and the literature shows evidence of its diagnostic effectiveness. HSV allows for real-time visualization of the mucosal vibration of the vocal folds and possible visualization of glottic closure irregularities during phonation. Moreover, it allows for the extraction of digital videokymography (DKG) parameters^(5,6)^.

DKG is an instrument that allows for direct vibratory observation of the vocal fold vibration patterns and quantitative analysis of the glottic function on a horizontal plane of observation^(7)^. DKG evaluation provides the detection of laryngeal asymmetries, amplitude of mucosal wave propagation, and glottal closure information^(8-10)^. Its analysis looks at the maximum, minimum, and mean amplitude of vocal fold vibration, each vocal fold's frequency, and closed quotient, which is a measure of glottal closure^(11)^.

Diagnostic accuracy is of fundamental importance to improve the treatment of patients with dysphonia. Research using HSV and DKG are promising since they are technologies that allow the visualization of the glottic cycle in a real and static way, and with a midline selection point that allows for analysis and understanding of the dynamics of voice production^(12)^.

Composite indicators or numerical indices are tools that enable the synthesis of variables related to a particular event^(13)^. In light of the voice's multidimensional characteristics, the main variables used in the characterization of these indices are auditory-perceptual analysis (sustained vowel phonation, connected speech, and phonetically balanced text), and acoustics, aerodynamics, and respiratory analyses^(14-17)^.

Numerical indices are instruments that can be applied in the voice clinic, during the evaluation and therapeutic processes, and used to monitor the evolution of the dysphonia, as a complementary method to vocal intervention. Among the most used indices are the *Dysphonia Severity Index* (DSI)^(14)^, the *Cepstral Spectral Index of Dysphonia* (CSID)^(15)^, the *Acoustic Voice Quality Index* (AVQI)^(16)^, and the *Acoustic Breathiness* Index (ABI)^(17)^, which take into consideration acoustic, cepstral, and aerodynamic analyses to determine dysphonia.

Improved laryngeal diagnostic accuracy can optimize voice therapy by helping to better define vocal techniques and improve voice treatment prognosis.

The purpose of this study was to develop a composite indicator called Videokymographic Index of Glottic Function – VIGF, from digital videokymography (DKG) parameters, captured by high-speed videoendoscopy (HSV) in women with and without behavioral laryngeal alterations. VIGF could help further improve the diagnostic accuracy of voice disorders, as videokymography is composed of several parameters and its analysis provides different information about the glottal cycle.

## METHODS

This is a retrospective study for the construction of a Composite Indicator, which followed all the steps recommended by the *Standards for Reporting Diagnostic Accuracy Studies* (STARDS)^(17)^. This research was approved by the Research Ethics Committee from the Federal University of Minas Gerais (UFMG), protocol number CAAE 44848115.0.0000.5149.

This study used a convenience sample of 92 women, who were divided into two groups. Group 1 (G1) was composed of women with laryngeal alterations, altered voice quality, and presence of vocal complaints. Inclusion criterion for G1 participants was having a diagnosis of behavioral dysphonia^(18)^. Group 2 (G2) was composed of women with no laryngeal alterations, with neutral voice quality, and no vocal complaints. Data was extracted from the high-speed videoendoscopy (HSV) database of the Functional Health Observatory (OSF/UFMG) from 2017 to 2018. Participants were contacted by telephone and asked about their agreement to carry out this research, as this was a database analysis.

Inclusion criteria for laryngeal images selection included having women between 18 and 45 years of age. Inclusion criteria for G1 was a diagnosis of behavioral dysphonia that included an altered laryngeal exam with the presence of vocal fold tissue or structural alterations affecting its vibration patterns and/or glottal closure, altered auditory-perceptual voice quality, and presence of vocal complaints^(18)^. Inclusion criteria for G2 included a laryngeal exam without any alterations, a neutral voice quality, and no vocal complaints. Exclusion criteria included being pregnant or being in a menstrual or premenstrual period on the day of the examination. Exclusion criteria further included women who presented with exacerbated nausea reflex, self-reported trauma or surgery in the cervical region, laryngeal or infectious diseases of the upper airways, hormonal diseases, or low sharpness of HSV images.

Laryngeal diagnoses were obtained via HSV by two otolaryngologists with more than five years of experience. Each exam consisted of 2,000 recorded images per second, using a 70° rigid laryngoscope with a 300W xenon light (KayPentax®, Lincoln Park, New Jersey) with a color high-speed videoendoscopy system model 9710. The image resolution used was 512 x 512 pixels with 8 bit RGB color mode. Laryngeal images were obtained by recording a sustained vowel /i/, at a habitual pitch and loudness. The best sequence of images was selected after the beginning of the vowel emission. Data from the midpoint of the glottis was analyzed, as the most prevalent lesions typically occur at the mid-third portion of the vocal fold vibration pattern^(19,20)^.

Laryngeal images recorded and selected for the research were evaluated by the two otolaryngologists, and the Woo^(21)^ classification was used to diagnose the type of glottal closure phase.

All participants' voices were recorded in an acoustically treated room for the auditory-perceptual voice quality assessment, and processed directly on a Dell® computer, model Optiplex GX260, with a professional sound card Direct Sound®, and using a unidirectional, condenser headset Shure® microphone at a two centimeters mic-to-mouth distance, with a directional pickup angle of 45°. Participants were asked to sustain the vowel /a/ at a habitual pitch and loudness for as long as possible, in a seated position. Voice recordings were obtained prior to performing the HSV.

Voice quality assessment was performed via auditory-perceptual analysis of the sustained vowel /a/ by three voice specialized speech-language pathologists. Voice classification was based on the general degree of dysphonia on a four-point scale, where zero indicated neutral voice quality, and three indicated intensely altered voice quality.

Vocal complaints were assessed on the day of the HSV. Voice self-assessment analysis (having/not having a good or very good voice) and analysis of the presence/absence of vocal symptoms (fatigue and/or discomfort) were performed. Vocal complaints were considered present as indicated by the voice quality self-perception and the presence of vocal symptoms.

A total of 112 laryngeal and voice image samples were analyzed, of which 92 images were included in this study. Group 1 (G1) consisted of 55 women with behavioral dysphonia, with a mean age of 29.01 years (SD=4.33). The main laryngeal alterations were as follow: Vocal nodules (N=24), vocal fold cyst (N=20), mid-posterior glottal gap (N=4), hourglass glottal gap (N=1), parallel glottal gap (N=1), vasculodysgenesis (N=4), and polypoid lesion (N=1). Voice quality in G1 were divided into mild (N=49) and moderate (N=6) altered voice quality, with presence of vocal complaint.

Group 2 (G2) consisted of 37 women with a normal larynx, with a mean age of 26.37 years (SD=3.87), presenting with no structural abnormalities on laryngeal examination. Only a posterior glottal chink was observed in G2, which was considered physiologically within normal limits for the subjects' gender^(2)^. All G2 participants presented with neutral voice quality and no vocal complaints.

Twenty laryngeal images in this study were not selected for the research because they did not meet the inclusion or exclusion criteria. Ten laryngeal images presented low sharpness resolution that made the DKG analysis unfeasible. Six patients showed signs of gastroesophageal reflux. Three patients reported a history of laryngeal microsurgery. One patient presented with an upper airway infection.

The quantitative analysis of the laryngeal images for DKG parameters extraction was performed using the KayPENTAX® image processing program called KIPS® (Kay's Image Processing Software), version 1.11.

### Digital videokymography (DKG) analysis

**Figure 1:**
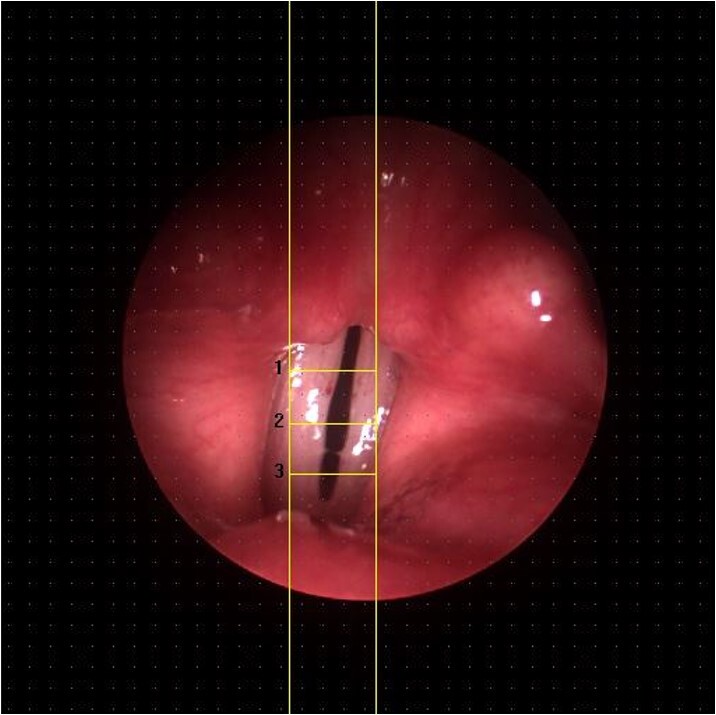
Manual positioning of vertical and horizontal lines for videokymographic assembly

To put together the videokymography on the KIPS®, the laryngeal image region was initially delimited manually for analysis. Two vertical lines were used to restrict the width of the videokymographic image and three horizontal lines were used to separate the posterior (line 1), middle (line 2) and anterior (line 3) thirds, determining the regions of the vocal fold vibration patterns for analysis (Figure 1). Line 2 results of the DKG were considered for this research development.

Recordings were selected for image analysis and trimmed, excluding the beginning and end of the videos. The program automatically performed a two-dimensional montage of the mucoondulatory movement of the vocal fold vibration patterns. The color of the image was converted to grayscale to extract the measurements. The edge detection rectangle adjustment tool and the edge detection tool were used to extract the measurements, and the glottic rhyme was selected for each determined line with the generated DKG chart. The KIPS® analyzed and quantified the DKG chart data, described in Table 1.

**Table 1:** Digital videokymography parameters of the larynx

**Parameters**	**Parameter Description**	**Measurement**
Minimum opening	Minimum vocal fold opening throughout the glottal cycle on the line selected by the kymograph, with zero indicating complete closure	Pixel (px)
Mean opening in the given region	Mean vocal fold opening throughout the glottal cycle on the line selected by the kymograph.	Pixel (px)
Maximum opening	Maximum vocal fold opening throughout the glottal cycle on the line selected by the kymograph	Pixel (px)
Right vocal fold amplitude	Amplitude of the right vocal fold opening on the line selected by the kymograph.	Pixel (px)
Left vocal fold amplitude	Amplitude of the left vocal fold opening on the line selected by the kymograph.	Pixel (px)
Right vocal fold frequency	Frequency of the right vocal fold opening on the line selected by the kymograph.	Pixel (px)
Left vocal fold frequency	Frequency of the left vocal fold opening on the line selected by the kymograph.	Pixel (px)
Closed Quotient	Percentage of the closed phase of the vocal fold. It is the ratio between the time of the closed phase and the total time of the glottal cycle, with zero indicating no glottal closure, and one indicating no glottal opening on the line selected by the kymograph.	Percentage (%)

Statistical analysis was performed using the MINITAB statistical program version 19. A descriptive analysis of the data was performed first with measures of central tendency and dispersion. Logistic regression was performed for the elaboration of the VIGF, together with the Hosmer-Lemeshow Test^(22)^, which compared the frequency of events observed and signaled by the logistic regression model.

For the Hosmer-Lemeshow Test^(22)^, the 92 examined women were divided into 10 groups according to the magnitude of the probabilities of vocal alteration indicated by the logistic regression model. The number of women with laryngeal alterations per group were then compared to the expected number signaled by the model. In a similar way, the women with no laryngeal alterations were compared to the alterations observed and signaled by the model. The closer the observed values were to those indicated by the logistic regression model, the better the fit. The Chi-Square distribution proposed by Hosmer-Lemeshow^(22)^ evaluated this proximity.

The significance level of each variable was also assessed simultaneously to the Hosmer-Lemeshow test^(22)^ by using the likelihood ratio test available in the XLSTAT statistical software. A valid logistic regression model must have a high p-value in the Hosmer-Lemeshow test (usually >5% significance) and low p-values for the model variables related tests (usually <5% significance).

The ROC curve was obtained using the XLSTAT software to assess sensitivity and specificity.

## RESULTS

**Figure 2:**
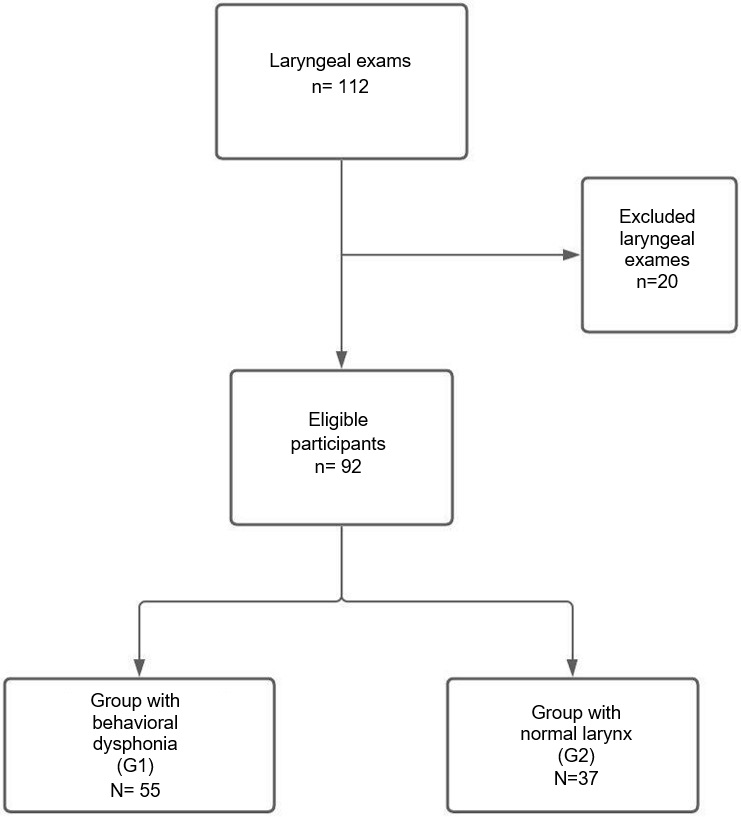
Sequence diagram with the research participants' flow

Figure 2 showed the flow of participants through a sequence diagram, according to the STARDS standards^(17)^.

A descriptive analysis of the variables was initially performed through measures of central tendency and dispersion, as seen in Table 2. G1 subjects presented with lower mean values in the parameters of maximum and mean DKG openings; right and left vocal fold amplitude; closed quotient; and higher mean values of the opening frequency parameters of the right and left vocal folds, characterizing a glottal cycle with less mucoondulatory movement excursion and with a greater frequency of vibration of the vocal fold vibration patterns.

**Table 2:** Descriptive analysis of the subjects' videokymographic parameters

***G1 (N=55)***					
***Parameter***	***Max***	***Min***	***Mean***	***Median***	***SD***
Maximum Opening	28.00	0.00	14.30	15.00	6.18
Mean Opening	17.33	0.00	7.45	6.78	4.92
L Frequency	5.06	0.00	1.95	1.92	1.38
L Amplitude	398.40	31.30	228.40	218.80	99.60
R Frequency	6.15	0.00	2.13	1.78	1.61
R Amplitude	437.50	31.30	228.10	218.80	104.50
Closed Quotient	100.45	0.00	30.91	30.80	29.01
***G2 (N=37)***					
***Parameter***	***Max***	***Min***	***Mean***	***Median***	***SD***
Maximum Opening	41.00	11.00	22.08	20.00	8.09
Mean Opening	20.00	1.87	8.84	8.64	4.50
L Frequency	312.5	198.03	202.17	218.75	55.58
L Amplitude	6.9	1.53	3.67	3.35	1.68
R Frequency	343.75	198.07	201.78	218.75	66.26
R Amplitude	8.83	1.44	4.20	3.80	1.91
Closed Quotient	67.50	10.00	41.53	43.41	11.83

Caption: G1: Women with altered laryngeal examination and vocal quality; G2: Women with normal laryngeal examination and neutral voice quality; Max: maximum value; Min: minimum value; Mean: Mean value; SD: Standard Deviation; L: Left; R: Right

Logistic regression was used to understand the classification process of the participants. Participants were classified as belonging to either group G1 or G2 and such classification depended on the variables described in Table 2. In the first stage of logistic regression, all variables described in Table 2 were analyzed, as represented by the following Equation 1.1:


$$P\left(Y=1\right)=\frac{e^{\left(\beta_{0}+\beta_{1}X_{1}+\beta_{2}X_{2}+\beta_{3}X_{3}+\beta_{4}X_{4}+\beta_{5}X_{5}+\beta_{6}X_{6}+\beta_{7}X_{7}\right)}}{1-e^{\left(\beta_{0}+\beta_{1}X_{1}+\beta_{2}X_{2}+\beta_{3}X_{3}+\beta_{4}X_{4}+\beta_{5}X_{5}+\beta_{6}X_{6}+\beta_{7}X_{7}\right)}}$$ (1.1)

$P\left(Y=1\right)$ indicated the probability of behavioral laryngeal alterations, conditioning the variables X1,…,X7 described in Table 3.

**Table 3:** Logistic regression analysis of videokymographic parameters of groups of women with and without laryngeal alteration

Variables	Value	Inferior limit	Superior limit	p-value
Intercept	7.8563	3.3547	12.3579	**0.0006**
Maximum opening (*X_1_* )	-0.2135	-0.3777	-0.0493	**0.0108**
Mean opening (*X_2_* )	-0.037	-0.2531	0.1791	0.7372
LVF Amplitude (*X_3_* )	0.0095	-0.5337	0.5527	0.9727
LVF Frequência (*X_4_* )	-0.0023	-0.016	0.0115	0.7472
RVF Amplitude (*X_5_* )	-0.3044	-0.8339	0.225	0.2598
RVF Frequency (*X_6_* )	0.0031	-0.0102	0.0163	0.6508
Closed Quotient (*X_7_* )	-0.0706	-0.1169	-0.0244	**0.0028**

Caption: Bold: significance level (p<0,05); LVF: Left vocal fold; RVF: Right vocal fold

The statistically significant variables were maximum opening and closed quotient, according to the likelihood ratio test, at a significance level of 5% (α). The Hosmer Lemeshow test^(22)^ verified the quality of fit and showed a well-adjusted model (p-valor=0,794). The intercept $\beta_{0}$ is an auxiliary parameter used to calculate the probability of change when, theoretically, all explanatory variables assumed the value zero. In mathematical terms, this probability was represented by $\frac{e^{\beta_{0}}}{1-e^{\beta_{0}}}$.

In the second stage of logistic regression, the selected variables (maximum opening and closed quotient) were analyzed to create the Videokymographic Index of Glottic Function (VIGF). Table 4 showed the description of the data. The Hosmer Lemeshow Test^(22)^ confirmed a goodness of fit with a p-value=0.198. The likelihood ratio tests indicated a significance of the maximum opening and closed quotient variables (p-value ≅0) as well as the need for intercept. Aditionally, McFadden's R^2^ value^(23)^ obtained with the XLSTAT software resulted in a value of 0.384. According to Louviere et al.^(23)^, McFadden's R^2^ values between 0.2 and 0.4 are indicative of an optimal logistic regression model fit.

**Table 4:** Final logistic regression analysis of videokymographic parameters of groups of women with and without laryngeal alteration

Variables	Value	Inferior Limit	Superior Limit	p value
Intercept	8.1318	4.7873	11.4763	**< 0.0001**
Maximum opening	-0.2964	-0.4238	-0.1690	**< 0.0001**
Closed Quotient	-0.0703	-0.1061	-0.0344	**0.0001**

Caption: Bold: significance level (p<0.05)

For the validity of the final model obtained in this second phase, the metric value “events per variable” (*EPV*)^(24)^ showed a result of 37/2=18.5. This calculation took into consideration the “no alterations” event, as it included a smaller amount of subjects with no alterations (37 women), compared to subjects presenting with alterations (55 women). The value 2 in the EPV result indicated the number of variables in the final model (maximum opening and closed quotient). According to the literature^(24)^, a sample size in a logistic regression model must have an EPV≥10 in order to properly estimate the parameters. Therefore, the sample size of this study was judged to be adequate as the resulting EPV of the final model presented in this work was 18.5^(25)^.

The VIGF cut-off value obtained by the logistic regression model was 0.71.

The probability of success obtained from the ROC curve analysis was 81.5%. Sensitivity was 76.4%, which was the probability of judging a woman to be with a behavioral dysphonia as having an altered laryngeal presentation while actually having an altered laryngeal examination. Specificity was 89.2%, which was the probability of judging a woman as having a normal laryngeal presentation while actually not presenting with any laryngeal alterations on examination.

**Figure 3:**
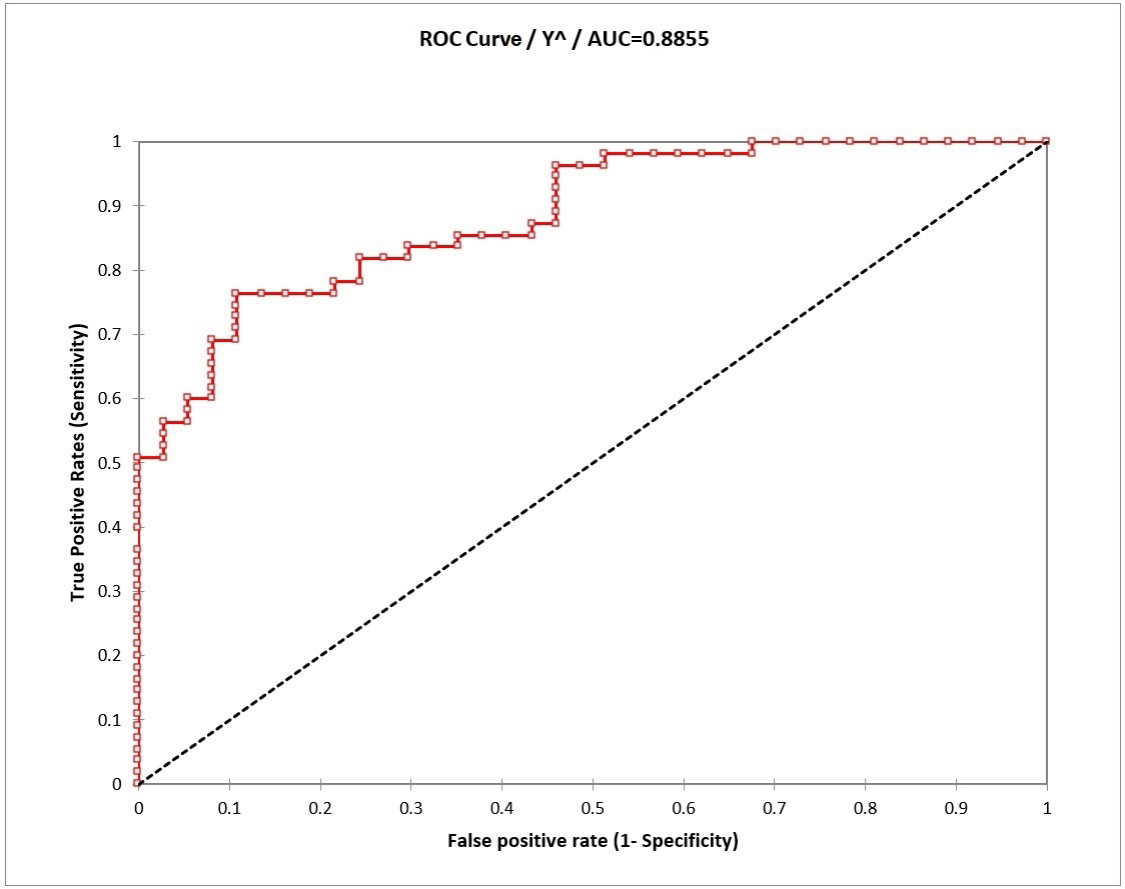
ROC curve to illustrate the VIGF's accuracy

The area under the ROC curve (*Receiver Operating Characteristic*) was approximately 0.89 indicating an excellent accuracy level^(24,25)^. Additionally, the area under the ROC curve was rejected to be 0.5 (random pattern) with a significance level of 5% with a p-value < 0.0001, which can be seen in Figure 3.

Therefore, the VIGF can be described below by the logistic regression model, where *e* represented the exponential function, *MaxOp* the maximum opening value and *ClQuo* represents the closed quotient values (Equation 1.2).


$$VIGF=\frac{e^{8,1318-0,2964MaxOp-0,0703ClQuo}}{1-e^{8,1318-0,2964MaxOp-0,0703ClQuo}}$$ (1.2)

VIGF values ≥0.71 indicated a high probability of laryngeal alteration occurrence in cases of behavioral dysphonia, while values <0.71 indicated a high probability of normal larynges in women.

## DISCUSSION

The purpose of this study was to develop the VIGF as a laryngeal diagnosis instrument for women with behavioral dysphonia. The instrument ​​proved to be an evaluation method with adequate sensitivity and specificity values and with a probability of success of 81.5%.

Direct observation of vocal fold vibration patterns and laryngeal structures is essential for the diagnosis of voice disorders^(26)^. HSV is an instrument capable of overcoming limits of traditional laryngoscopy. It provides data on the real-time glottal cycle behavior, allowing for a more effective and visual clinical diagnosis, with an easily interpretable analysis^(27)^.

HSV, paired with DKG in clinical practice, substantially enriches the possibilities of laryngeal diagnosis, and quantitatively reveals the dynamic behavior of the vocal folds and other laryngeal structures^(28)^. This method also allows to characterize in greater detail irregular vibrations, glottal cycle duration, mucosal wave, vertical relationship, and glottal closure pattern^(28)^.

Based on the analysis of the glottic cycle of 252 participants, of both sexes, HSV presented 100% diagnostic accuracy in moderate-grade vocal alterations and 64% in severe-grade alterations when compared to videoendoscopy^(29)^. Patients with and without laryngeal alterations were evaluated by both methods, and the authors observed significant differences in the classification of vocal conditions, since HSV is able to provide more accurate images of phonation over many glottic cycles, favoring the quality of the diagnosis^(30)^.

Health indices are used to measure and assign values that define standards, providing comparisons and improving diagnostic accuracy. These indices are widely used in other professional areas and help understand and create reference values for clinical practice, based on the observation of the parameters that best concisely describe the event^(31)^.

The growing interest in the clinical quantification of voice quality led researchers to create simple numerical indices using techniques of acoustic and auditory-perceptual analyses. There are voice analysis indices described in the literature^(14-16,18)^, based on sustained vowel and connected speech.

Researchers found sensitivity and specificity values at 82.4% and 92.9% respectfully in the evaluation of vocal breathiness, proposed by the *Acoustic Breathiness Index* (ABI)^(18)^. Sensitivity and specificity values of 85% and 100% were respectively found in the evaluation of voice quality, based on acoustic measures proposed by the *Acoustic Voice Quality Index* (AVQI)^(16)^, which provided precise parameters for the identification and separation of normal and dysphonic voices.

CSID^(15)^ dysphonia severity screening of 322 subjects, through the use of cepstral measurements, presented a sensitivity of 75% and specificity of 73%, with a probability of success of 85%. The Dysphonia Severity Index (DSI)^(14)^ presented sensitivity and specificity values of 72% and 75% respectively, for the determination of the general degree of dysphonia.

The use of systematic recommendations (or guidelines) for the construction of clinical practices are widely spread, in order to systematize information on certain health conditions^(17)^. Parameters such as sensitivity and specificity are essential to correctly determine positive and negative cases, which are measured using standard tests. This study chose to use the STARD to establish construction criteria, thus reducing possible biases^(17)^.

The diagnostic accuracy of a clinical test is linked to its sensitivity and specificity values. The VIGF had a cutoff value of 0.71, sensitivity of 76.4%, specificity of 89.2%, with a probability of success of 81.5%. The determination of these values was essential to establish the diagnostic power of the instrument. The VIGF results showed high sensitivity and specificity values, which determine the predictive capacity of this diagnostic test^(15)^. These results were further confirmed by the 89% ROC Curve area, which featured excellent discrimination power^(22)^ between women with a normal laryngeal presentation and women with altered laryngeal presentation due to behavioral dysphonia. Other studies^(16,18)^ also used the ROC Curve to determine the specificity and sensitivity in the diagnostic process of an instrument, presenting values that varied between 0.5 to 0.68. Results obtained through the ROC curve of this study reinforced the VIGF as a sensitive and accurate instrument in the laryngeal evaluation of women with behavioral dysphonia.

The VIGF described in this study can be considered a differential in the quality of laryngeal diagnosis in women with behavioral dysphonia, because it used the most recent laryngeal evaluation technology available.

One of the limitations of this study was the use of only female subjects, who presented with behavioral laryngeal lesions.

All laryngeal image and voice quality assessments were carried out by consensus by field experts with the aim of a collaborative diagnostic evaluation process. Other diagnostic evaluations were possible and included, for example, analysis of the most prevalent results of evaluators with higher levels of agreement. Future studies are necessary to improve understanding of the best evaluation and classification strategies of subjective analyses, such as the visual-perceptual analysis of laryngeal images, and the auditory-perceptual voice analysis.

The *American Speech Language Hearing Association* (ASHA)^(2)^ recommended instrumental voice assessment protocols, including laryngeal image assessment, acoustic voice analysis, and aerodynamic assessment. The objective of the protocols was to define vocal assessment markers, allow comparisons between different clinical and research centers, and facilitate the evaluation of the effectiveness of voice treatment^(2)^. ASHA's recommendations for laryngeal evaluation are videolaryngoscopy for structural laryngeal analysis, and videolaryngostroboscopy for analysis of the vibratory movement of vocal fold vibration patterns^(2)^. The literature reinforces videolaryngostroboscopy as the gold standard for the diagnosis of vocal alterations^(32)^, and HSV can be complementary to stroboscopy, mainly for dysphonia characterized by asymmetrical or aperiodic vibrations of the vocal fold vibration patterns^(32)^. HSV is a recent technology, dependent on a high-cost instrument, and still with little access to laryngology clinics. However, HSV research remains important in order to analyze the real diagnostic value of this procedure.

Laryngeal assessment is typically within the scope of practice of otolaryngologists. However, ASHA^(2)^ recognizes speech-language pathologists' knowledge and skills in performing and interpreting functional analyses of laryngeal images, as an aid in making the best clinical decision for the therapeutic treatment of dysphonia.

Future VIGF research is needed and is important in order to assess the instrument's application in clinical voice practices.

## CONCLUSION

The VIGF presented a cutoff value of 0.71, where values ≥0.71 represented the presence of laryngeal alterations in cases of behavioral dysphonia, while values <0.71 represented normal laryngeal presentations. The probability of success was 81.5%, sensitivity was 76.4% and specificity was of 89.2%. The VIGF can be considered a tool used to optimize the laryngeal diagnosis and to better define voice therapy treatment for behavioral dysphonia, thus enhancing the prognosis in the voice clinic.
