# Supplementary material for: Videokymographic index of glottic function: an analysis of diagnostic accuracy
Source: Codas. 2022 Oct 17;35(1):e20210214. doi: 10.1590/2317-1782/20212021214en (PMC10010432; doi:10.1590/2317-1782/20212021214en)
Supplement: Translation

# Translation: Índice videoquimográfico da função glótica: análise da precisão diagnóstica

**DOI:** 10.1590/2317-1782/20212021214pt

**Authors:** Alice Braga de Deus, Roberto da Costa Quinino, Marco Aurélio Rocha Santos, Ana Cristina Côrtes Gama

**Affiliations:** 1 Programa de Pós-graduação em Ciências Fonoaudiológicas, Faculdade de Medicina, Universidade Federal de Minas Gerais – UFMG - Belo Horizonte (MG), Brasil.; 2 Departamento de Estatística, Instituto de Ciências Exatas, Universidade Federal de Minas Gerais – UFMG - Belo Horizonte (MG), Brasil.; 3 Hospital das Clínicas, Universidade Federal de Minas Gerais – UFMG - Belo Horizonte (MG), Brasil.; 4 Departamento de Fonoaudiologia, Faculdade de Medicina, Universidade Federal de Minas Gerais – UFMG - Belo Horizonte (MG), Brasil.

**Keywords:** Quimografia, Voz, Disfonia, Aprovação de Teste de Diagnóstico, Laringe

## Abstract

**Objetivo:**

Elaborar um indicador composto denominado Índice Videoquimográfico da Função Glótica – IVFG, a partir de parâmetros da videoquimografia digital, captados pelo exame de videolaringoscopia de alta velocidade de mulheres sem e com alterações laríngeas de etiologia comportamental.

**Método:**

A amostra foi composta por 92 mulheres, destas 55 apresentaram disfonia comportamental, com presença de alterações laríngeas e vocais, e 37 mulheres sem alterações laríngeas e vocais, entre 18 a 45 anos. A avaliação vocal foi realizada por consenso pela análise perceptivo-auditiva da vogal /a/ em frequência e intensidade habituais, e classificação através do grau geral da disfonia, onde G0 indicou qualidade vocal neutra e G1 a 3 qualidade vocal alterada. As imagens laríngeas foram obtidas pela gravação da emissão da vogal /i/, em frequência e intensidade habituais para análise da videoquimografia digital. A construção do IVFG se deu pela escolha do ponto médio da glote para análise e, elaboração foi realizada regressão logística pelo programa MINITAB 19.

**Resultados:**

A regressão logística contou com duas etapas, sendo que a etapa 1 constou da análise de todas as variáveis, onde as variáveis abertura máxima e fechamento glótico apresentaram significância estatística (p-valor <0.05) e o modelo se encontrou bem ajustado de acordo com o teste de Hosmer-Lemeshow (p-valor=0,794); na etapa 2, as variáveis selecionadas foram novamente analisadas e o modelo também se mostrou bem ajustado (p-valor=0,198). O IVFG foi definido por IVFG=e^(8,1318-0,2941AbMax-0,0703FechGlo)/1+e^(8,1318-0,2941AbMax-0,0703FechGlo).

**Conclusão:**

O IVFG apresenta valor de corte igual a 0,71. A probabilidade de acerto é de 81,5%, sensibilidade 76,4%, especificidade de 89,2%.

## INTRODUÇÃO

A voz se constitui como uma principal ferramenta da comunicação, transmitindo importantes características do falante^(1)^. Sabe-se que a fonação é coordenada por mecanismos neurofisiológicos, os quais exigem harmonia e integridade do aparelho fonador^(1)^.

A classificação das disfonias pode ser baseada em diversos critérios, uma vez que a voz é o resultado da integração de múltiplas dimensões. As disfonias comportamentais são aquelas que a etiologia é dada pelo comportamento vocal^(2)^. Lesões com alterações teciduais de mucosa, como nódulos vocais, edemas, pólipos, dentre outros, exemplificam este quadro de disfonia^(1)^.

Nas disfonias comportamentais a presença de alteração vocal associada com sintomas de esforço fonatório podem ser fatores limitadores da qualidade de vida do paciente disfônico^(3)^. O diagnóstico desta alteração é multidisciplinar, onde os exames laríngeos serão instrumentos fundamentais na descrição do padrão vibratório das pregas vocais (PPVV) e do comportamento dos ciclos glóticos^(4)^.

A avaliação otorrinolaringológica dispõe de equipamentos com tecnologias variadas, sendo a videolaringoestroboscopia o método que permite a visualização do movimento muco-ondulatório da mucosa das PPVV^(4)^. A literatura evidencia a eficácia diagnóstica da videolaringoscopia de alta velocidade (HSV), instrumento capaz de captar 2000 a 6000 imagens por segundo, permitindo a real visualização da vibração da mucosa das PPVV e possíveis irregularidades glóticas durante a fonação, além de possibilitar a extração dos parâmetros da videoquimografia digital (DKG)^(5,6)^.

Considerando o plano horizontal de observação, a DKG é um instrumento que permite a observação direta da vibração das PPVV e análise quantitativa da função glótica^(7)^. Sua avaliação permite a detecção de assimetrias laríngeas, amplitude de propagação da onda mucosa e fechamento da glote^(8-10)^. Em sua análise, considera as variáveis abertura máxima, mínima e média, amplitude de vibração e frequência de cada prega vocal, além de avaliar o fechamento glótico^(11)^.

A precisão diagnóstica é de fundamental importância para o aperfeiçoamento no tratamento de pacientes com disfonia. Pesquisas que utilizam a HSV e a DKG são promissoras, posto que são tecnologias que permitem a visualização do ciclo glótico de forma real, estática e com ponto de seleção na linha média, possibilitando o conhecimento e compreensão sobre a dinâmica da produção vocal^(12)^.

Os Indicadores compostos ou índices numéricos são ferramentas que possibilitam a síntese de variáveis relacionadas a um determinado evento^(13)^. Considerando o caráter multidimensional da voz, as principais variáveis utilizadas na caracterização destes índices são análise perceptivo-auditiva (vogal sustentada, fala encadeada e texto balanceado foneticamente), acústica, aerodinâmica e respiratória^(14-17)^.

Na clínica vocal, os índices são instrumentos que podem ser aplicados na avaliação, processo terapêutico e monitoramento da evolução do quadro disfônico, sendo um método complementar à intervenção vocal. Dentre os índices mais utilizados, citam-se o *Dysphonia Severity Index* (DSI)^(14)^, *Cepstral Spectral Index of Dysphonia* (CSID)^(15)^, *Acoustic Voice Quality Index* (AVQI)^(16)^, *Acoustic Breathiness* Index (ABI)^(17)^, os quais consideram as variáveis acústicas, cepstrais, e aerodinâmicas em suas análises para determinação da disfonia.

O objetivo desse estudo é elaborar um indicador composto denominado Índice Videoquimográfico da Função Glótica – IVFG, a partir de parâmetros da videoquimografia digital, captados pelo exame de videolaringoscopia de alta velocidade de mulheres sem e com alterações laríngeas de etiologia comportamental.

O desenvolvimento da tecnologia da semiologia laríngea propiciou a avaliação quantitativa das fases do ciclo glótico, portanto, o Índice Videoquimográfico da Função Glótica (IVFG) beneficiará a precisão diagnóstica, uma vez que a videoquimografia é composta por diversos parâmetros e sua análise fornece diferentes informações do ciclo glótico. A melhor precisão diagnóstica laríngea otimiza o tratamento fonoterápico, por meio do auxílio na definição de melhores técnicas vocais, melhorando o prognóstico do tratamento vocal.

## MÉTODO

Trata-se de um estudo retrospectivo para a construção de um Indicador Composto, que seguiu todas as etapas recomendadas pelo *Standards for Reporting Diagnostic accuracy studies* (STARDS)^(17)^. Esta pesquisa foi aprovada pelo Comitê de Ética em Pesquisa (COEP) da Universidade Federal de Minas Gerais, sob o número CAAE 44848115.0.0000.5149.

Este estudo utilizou uma amostra de conveniência composta por 92 mulheres, que foram divididas em dois grupos. O Grupo 1 (G1) foi composto por mulheres com alterações laríngeas, alteração da qualidade vocal e presença de queixas vocais. Todas as participantes do G1 preencheram os critérios para o diagnóstico de disfonia de base comportamental^(18)^. O Grupo 2 (G2) foi formado por mulheres sem alterações laríngeas, qualidade vocal neutra e ausência de queixa vocal. Os dados analisados foram provenientes do banco de dados de imagens laríngeas da videolaringoscopia de alta velocidade (HSV) do Laboratório de Saúde Funcional em Fonoaudiologia (OSF/UFMG) no período de 2017 a 2018. Por se tratar da análise de um banco de dados, os participantes foram contatados por telefone e questionados sobre a concordância na realização desta pesquisa, não sendo necessária a assinatura de outro Termo de Consentimento Livre e Esclarecido (TCLE).

Para seleção das imagens laríngeas, foram critérios de inclusão ser mulher de 18 a 45 anos, e para o G1 apresentar exame laríngeo alterado com presença de alterações teciduais ou estruturais das PPVV e/ou de coaptação glótica; qualidade vocal alterada; e presença de queixa vocal, definindo um diagnóstico de disfonia de base comportamental^(18)^. Para o G2 foram critérios de inclusão apresentar exame sem alterações laríngeas, qualidade vocal neutra e ausência de queixa vocal. Os critérios de exclusão considerados foram, no dia da realização do exame, estar grávida ou em período menstrual ou pré-menstrual. Foram também excluídas mulheres que apresentaram reflexo de náusea exacerbado, autorreferiram traumas ou cirurgias na região cervical, doenças laríngeas ou infecciosas das vias aéreas superiores, doenças hormonais e imagens da HSV com baixa nitidez.

Para a definição do diagnóstico laríngeo foram realizadas avaliações com a HSV por dois médicos otorrinolaringologistas com experiência maior que cinco anos. Em cada exame foram gravadas 2000 imagens por segundo usando laringoscópio rígido de 70° com 300W de luz xênon (KayPentax ®, Lincoln Park, New Jersey) com sistema de videolaringoscopia de alta velocidade colorido modelo 9710. A resolução da imagem utilizada foi 512 x 512 pixels com modo de cor de 8 bit RGB. As imagens laríngeas foram obtidas pela gravação da emissão da vogal /i/, em frequência e intensidade habituais, sendo selecionada a melhor sequência de imagens após o início das emissões, previamente estabelecidos pelos autores. Durante a análise, optou-se pela escolha de interpretação dos dados referentes ao ponto médio da glote, uma vez que as lesões que acometem o terço médio das PPVV são as mais prevalentes^(19,20)^.

As imagens laríngeas gravadas e selecionadas para a pesquisa foram avaliadas pelos dois otorrinolaringologistas, e para o diagnóstico do tipo de fechamento glótico se utilizou a classificação de Woo^(21)^.

Para a avaliação perceptivo-auditiva da qualidade vocal, as vozes de todas as participantes foram gravadas em uma sala acusticamente tratada e foi processada diretamente em um computador da marca Dell®, modelo Optiplex GX260, com placa de som profissional marca Direct Sound® com a utilização de um microfone unidirecional, condensador, da marca Shure®, posicionado a dois centímetros da comissura labial, com ângulo de captação direcional de 45°. Foi solicitado à participante que, em posição sentada, emitisse a vogal sustentada /a/ de forma habitual e plena. As gravações das vozes ocorreram antes da realização do HSV.

A avaliação da qualidade vocal foi realizada por consenso por três fonoaudiólogos especialistas em voz, por meio da análise perceptivo-auditiva da vogal /a/ sustentada. A classificação vocal se deu através do grau geral da disfonia em uma escala de quatro pontos, onde zero indicou qualidade vocal neutra, e três qualidade vocal alterada de grau intenso.

A presença de queixa vocal foi avaliada no dia da realização da HSV, a partir análise da autopercepção da qualidade vocal (referir ter/não ter voz boa ou muito boa) e análise da presença/ausência de sintomas vocais (fadiga e/ou desconforto). A presença de queixa vocal foi definida pela autopercepção negativa da qualidade vocal e pela presença de sintomas vocais.

Do total de 112 amostras de imagem laríngea e voz analisadas, foram incluídas neste estudo 92 imagens. O Grupo 1 (G1) foi composto por 55 mulheres com idade média de 29,01 anos (DP=4,33). As principais alterações laríngeas encontradas foram nódulos vocais (N=24), cisto de prega vocal (N=20); fendas médio-posterior (N=4), dupla (N=1), e paralela (N=1); vasculodisgenesia (N=4); e lesão polipóide (N=1), além de qualidade vocal alterada de grau leve (N=49) e moderado (N=6), e presença de queixa vocal. O G1 caracterizou, portanto, o grupo de mulheres com disfonia comportamental.

O Grupo 2 (G2) foi composto por 37 mulheres com idade média de 26,37 anos (DP=3,87). As participantes deste grupo apresentaram exame laríngeo sem alterações estruturais. Observou-se somente presença de fenda triangular posterior nas mulheres, sendo esta considerada fisiológica^(2)^. Todas as participantes deste grupo apresentaram qualidade vocal neutra, e não possuíam queixa vocal. O G2 formou o grupo de mulheres com laringe normal.

Neste estudo 20 imagens laríngeas não foram selecionadas para a pesquisa por não atenderem os critérios de inclusão ou exclusão. Dez imagens laríngeas apresentaram baixa nitidez que inviabilizou a análise da DKG; seis pacientes apresentaram sinais de refluxos gastroesofágico; três pacientes referiram histórico de microcirurgia laríngea; e um apresentou infecção de vias aéreas superiores.

Para a extração dos parâmetros da DKG, a análise quantitativa das imagens laríngeas foi realizada por meio do programa de processamento de imagens denominado KIPS® (Kay's Image Processing Software), versão 1.11, fornecido pela KayPENTAX®.

### Análise da videoquimografia digital (DKG)

Para montagem do videoquimograma no KIPS®, o avaliador inicialmente delimitou manualmente a região da imagem laríngea para análise. Duas linhas verticais possibilitaram restringir a largura da imagem quimográfica e três linhas horizontais foram utilizadas para separar os terços posterior (linha 1), médio (linha 2) e anterior (linha 3), determinando as regiões das PPVV para análise (Figura 1). Para o desenvolvimento desta pesquisa foram considerados os resultados da DKG da linha 2.

**Figura 1:** Posicionamento manual das linhas verticais e horizontais para montagem do videoquimograma

Para análise da imagem foi selecionado o trecho médio das gravações, excluindo o início e o final dos vídeos. O programa executou a montagem bidimensional do movimento mucoondulatório das PPVV de forma automática. O avaliador converteu a cor da imagem para escala de cinza para extração das medidas, e para tal, utilizou a ferramenta de ajuste de retângulo de detecção de borda e da ferramenta de detecção de bordas, concluindo a seleção da rima glótica para cada linha determinada, com o gráfico da DKG gerado. O KIPS® analisou e quantificou os dados do gráfico da DKG, organizando-os em uma tabela. A descrição dos parâmetros está descrita na Tabela 1.

**Tabela 1:** Parâmetros da videoquimografia digital da laringe

**Parâmetros**	**Descrição dos parâmetros**	**Medida**
Abertura mínima	Indica a mínima abertura na linha selecionada pela videoquimografia. Zero indica o fechamento completo das pregas vocais	Pixel (px)
Abertura média na região determinada	Indica a abertura média na linha selecionada pela quimografia.	Pixel (px)
Abertura máxima	Indica a máxima abertura na linha selecionada pela videoquimografia.	Pixel (px)
Amplitude de abertura da prega vocal direita	Indica a amplitude da prega vocal direita durante a abertura, na linha selecionada pela videoquimografia.	Pixel (px)
Amplitude de abertura da prega vocal esquerda	Indica a amplitude da prega vocal esquerda durante a abertura, na linha selecionada pela videoquimografia.	Pixel (px)
Frequência de abertura da prega vocal direita	Medida de frequência predominante durante a abertura da prega vocal direita, na linha selecionada pela videoquimografia.	Pixel (px)
Frequência de abertura da prega vocal esquerda	Medida de frequência predominante durante a abertura da prega vocal esquerda, na linha selecionada pela videoquimografia.	Pixel (px)
Fechamento glótico	Representa a porcentagem da fase fechada da prega vocal. É a razão entre o tempo da fase fechada pelo tempo total do ciclo glótico. Neste caso, zero indica que não ocorre o fechamento glótico e um indica que não ocorre a abertura glótica na linha selecionada pela videoquimografia.	Porcentagem (%)

A análise estatística dos dados foi realizada pelo programa estatístico MINITAB versão 19. Primeiramente foi realizada uma análise descritiva dos dados com medidas de tendência central e dispersão. Para elaboração do IVFG foi realizada regressão logística, em conjunto com o Teste de Hosmer-Lemeshow^(22)^, o qual compara a frequência de acontecimentos de eventos observados e sinalizados pelo modelo de regressão logística.

A ideia do Teste de Hosmer-Lemeshow^(22)^ no contexto do presente trabalho implica em dividir as 92 mulheres examinadas em 10 grupos de acordo com a magnitude das probabilidades de alteração vocais indicadas pelo modelo de regressão logística. Em seguida comparamos para cada grupo o número observado de mulheres com alterações na laringe versus o número esperado sinalizado pelo modelo. De forma similar também comparamos a situação das mulheres sem alteração na laringe em relação as alterações observadas e sinalizadas pelo modelo. Quanto mais próximo for os valores observados daqueles indicados pelo modelo de regressão logística, melhor será o ajuste. Esta proximidade ou não é avaliada pelo distribuição Qui-Quadrado proposta por Hosmer-Lemeshow^(22)^.

Simultaneamente ao teste de Hosmer-Lemeshow^(22)^ também devemos avaliar a significância de cada variável no modelo. Para tanto utilizamos o teste da razão de verossimilhança disponível no software estatístico XLSTAT. Um modelo de regressão logística válido deve ter p-valor no teste de Hosmer-Lemeshow alto (usualmente maior que 5% - significância) e p-valores baixos para os testes relativos as variáveis do modelo (usualmente menor que 5% - significância).

Para avaliar a sensibilidade e a especificidade, a Curva ROC foi obtida por meio do software XLSTAT.

## RESULTADOS

Na Figura 2 se observa o fluxo dos participantes por meio de um diagrama de sequência, de acordo com as normas STARDS^(17)^.

**Figura 2:** Diagrama de sequência com o fluxo dos participantes da pesquisa

Inicialmente, foi realizada a análise descritiva das variáveis através das medidas de tendência central e de dispersão, de acordo com a Tabela 2. As mulheres com quadros de alteração laríngea por disfonia comportamental (G1) apresentam menores valores médios nos parâmetros da DKG de aberturas máxima e média; de amplitude de pregas vocais direita e esquerda; e de fechamento glótico; e de maiores valores da média dos parâmetros de frequência de abertura das pregas vocais direita e esquerda, caracterizando um ciclo glótico com menor excursão do movimento mucoondulatório e com maior frequência de vibração das PPVV.

**Tabela 2:** Análise descritiva dos parâmetros videoquimográficos das mulheres

***G1 (N=55)***					
***Parâmetro***	***Max***	***Min***	***Med***	***Mediana***	***DP***
Abertura máxima	28,00	0,00	14,30	15,00	6,18
Abertura média	17,33	0,00	7,45	6,78	4,92
Frequência de abertura E	5,06	0,00	1,95	1,92	1,38
Amplitude de abertura E	398,40	31,30	228,40	218,80	99,60
Frequência de abertura D	6,15	0,00	2,13	1,78	1,61
Amplitude de abertura D	437,50	31,30	228,10	218,80	104,50
Fechamento glótico	100,45	0,00	30,91	30,80	29,01
***G2 (N=37)***					
***Parâmetro***	***Max***	***Min***	***Med***	***Mediana***	***DP***
Abertura máxima	41,00	11,00	22,08	20,00	8,09
Abertura média	20,00	1,87	8,84	8,64	4,50
Frequência de abertura E	312,5	198,03	202,17	218,75	55,58
Amplitude de abertura E	6,9	1,53	3,67	3,35	1,68
Frequência de abertura D	343,75	198,07	201,78	218,75	66,26
Amplitude de abertura D	8,83	1,44	4,20	3,80	1,91
Fechamento glótico	67,50	10,00	41,53	43,41	11,83

Legenda: G1: Mulheres com exame laríngeo e qualidade vocal alterados; G2: Mulheres com exame laríngeo normal e qualidade vocal neutra; Max: valor máximo; Min: valor mínimo; Med: valor da média; DP: Desvio Padrão; E: Esquerda; D: Direita

Como as mulheres participantes do estudo foram classificadas pertencentes aos grupos G1 ou G2 e tal classificação podia depender das variáveis descritas na Tabela 2, optamos pelo uso da regressão logística para entender o processo classificatório. Em uma primeira etapa utilizamos a regressão logística com todas as variáveis descritas na Tabela 2 como representado pela Equação 1.1:


$$P\left(Y=1\right)=\frac{e^{\left(\beta_{0}+\beta_{1}X_{1}+\beta_{2}X_{2}+\beta_{3}X_{3}+\beta_{4}X_{4}+\beta_{5}X_{5}+\beta_{6}X_{6}+\beta_{7}X_{7}\right)}}{1-e^{\left(\beta_{0}+\beta_{1}X_{1}+\beta_{2}X_{2}+\beta_{3}X_{3}+\beta_{4}X_{4}+\beta_{5}X_{5}+\beta_{6}X_{6}+\beta_{7}X_{7}\right)}}$$ (1.1)

Em que $P\left(Y=1\right)$ indica a probabilidade de alterações laríngeas de etiologia comportamental condicionado as variáveis $X_{1},\ldots ,X_{7}$ descritas na Tabela 3.

**Tabela 3:** Análise da regressão logística dos parâmetros videoquimográficos dos grupos de mulheres sem e com alteração laríngea

Variáveis	Valor	Limite inferior	Limite superior	p-valor
Intercepto	7,8563	3,3547	12,3579	**0,0006**
Abertura máxima (*X_1_* )	-0,2135	-0,3777	-0,0493	**0,0108**
Abertura média (*X_2_* )	-0,037	-0,2531	0,1791	0,7372
Amplitude de abertura PVE (*X_3_* )	0,0095	-0,5337	0,5527	0,9727
Frequência de abertura PVE (*X_4_* )	-0,0023	-0,016	0,0115	0,7472
Amplitude de abertura PVD (*X_5_* )	-0,3044	-0,8339	0,225	0,2598
Frequência de abertura PVD (*X_6_* )	0,0031	-0,0102	0,0163	0,6508
Fechamento glótico (*X_7_* )	-0,0706	-0,1169	-0,0244	**0,0028**

Legenda: Negrito: nível de significância (p<0,05); PVE: prega vocal esquerda; PVD: prega vocal direita

Considerando um nível de significância 5% (α) as variáveis estatisticamente significantes, de acordo com o teste da razão de verossimilhança, foram abertura máxima e fechamento glótico. O Teste de Hosmer Lemeshow^(22)^, que tem por finalidade constatar a qualidade do ajuste, mostrou que o modelo está bem ajustado (p-valor=0,794). Como observado na Tabela 3, o intercepto $\beta_{0}$ é um parâmetro auxiliar para calcularmos a probabilidade de alteração quando, teoricamente, todas as variáveis explicativas assumirem o valor zero. Em termos matemáticos essa probabilidade é representada por $\frac{e^{\beta_{0}}}{1-e^{\beta_{0}}}$. A sua necessidade ou não é avaliada em termos de significância estatística.

Na segunda etapa da regressão logística, as variáveis selecionadas (abertura máxima e fechamento glótico) foram analisadas para criação do Índice Videoquimográfico da Função Glótica (IVFG). Na Tabela 4, observa-se a descrição dos dados. O Teste de Hosmer Lemeshow^(22)^ confirmou a bondade do ajuste apresentando *p-valor*=0,198 e os testes da razão de verossimilhança indicaram a significância das variáveis abertura máxima e fechamento glótico (p-valor ≅0) bem como da necessidade do intercepto. Adicionalmente, o valor do R^2^ de McFadden^(23)^ obtido com o software XLSTAT resultou em 0,384. De acordo com Louviere et al.^(23)^ valores de R^2^ de McFadden entre 0,2 e 0,4 é um indicativo de um ótimo ajuste do modelo de regressão logística.

**Tabela 4:** Análise final da regressão logística dos parâmetros videoquimográficos dos grupos de mulheres sem e com alteração laríngea

Variáveis	Valor	Limite inferior	Limite superior	p valor
Intercepto	8,1318	4,7873	11,4763	**< 0,0001**
Abertura máxima	-0,2964	-0,4238	-0,1690	**< 0,0001**
Fechamento glótico	-0,0703	-0,1061	-0,0344	**0,0001**

Legenda: Negrito: nível de significância (p<0,05)

Observamos também que, para validade do modelo final obtido nesta segunda fase, o valor da métrica “evento por variável” (*EPV*)^(24)^ apresentou o resultado 37/2=18,5. No cálculo usamos o evento “sem alteração”, pois a sua quantidade observada é menor, isto é, apresenta 37 mulheres enquanto a quantidade do evento “com alteração” foi de 55. O valor 2 indica a quantidade de variáveis no modelo final (abertura máxima e fechamento glótico). De acordo com a literatura^(24)^ um tamanho amostral necessário para estimar os parâmetros adequadamente em um modelo de regressão logística deve apresentar EPV≥10. Como o EPV do modelo final apresentado neste trabalho foi de 18,5, então o tamanho amostral pode ser julgado adequado para as conclusões obtidas^(25)^.

Neste momento, definiu-se o valor de corte para o IVFG igual a 0,71, de tal forma a maximizar o grau de acerto das mulheres pertencentes ao estudo nos grupos G1 ou G2, obtido pelo modelo de regressão logística.

A partir da análise da Curva ROC, a probabilidade de acerto geral encontrada foi de 81,5%, sensibilidade 76,4% (probabilidade de julgar uma mulher com laringe alterada decorrente de uma disfonia de etiologia comportamental quando ela realmente possui a laringe alterada) e especificidade de 89,2% (probabilidade de julgar uma mulher como laringe normal quando ela realmente não possui laringe alterada).

Destacamos também que a área sob a curva ROC (*Receiver Operating Characteristic*) foi de aproximadamente 0,89 indicando uma precisão excelente^(24,25)^. Adicionalmente rejeitamos que a área sob a curva ROC fosse 0.5 (padrão aleatório) com nível de significância 5% com um p-valor < 0,0001, o qual pode ser observado na Figura 3.

**Figura 3:** Curva ROC para ilustrar a acurácia do IVFG

Portanto, o IVFG pode ser descrito abaixo pelo modelo de regressão logística, onde *e* representa a função exponencial, *AbMax* o valor da abertura máxima e *FechGlo* representa valores do fechamento glótico (Equação 1.2).


$$IVFG=\frac{e^{8,1318-0,2964AbMax-0,0703FechGlo}}{1-e^{8,1318-0,2964AbMax-0,0703FechGlo}}$$ (1.2)

Para valores de IVFG ≥0,71 entendemos que existe alta probabilidade da ocorrência de alterações laríngeas em quadros de disfonia comportamental, enquanto valores <0,71 determinam alta probabilidade de laringes normais em mulheres.

## DISCUSSÃO

A elaboração do IVFG como instrumento de diagnóstico laríngeo de mulheres com disfonia comportamental foi possível, evidenciando ser um método de avaliação com adequados valores de sensibilidade, especificidade e com probabilidade de acerto de 81,5%.

A observação direta das PPVV e de estruturas laríngeas é essencial para o diagnóstico dos distúrbios da voz^(26)^. A HSV apresenta-se como um instrumento capaz de suprir os limites da laringoscopia tradicional, uma vez que fornece dados sobre o comportamento real do ciclo glótico, tornando o diagnóstico clínico mais eficaz e visual, sendo facilmente interpretável^(27)^.

Na prática clínica, a DKG associada a HSV enriquece substancialmente as possibilidades de diagnóstico laríngeo, e revela de forma quantitativa, o comportamento dinâmico das pregas vocais e de outras estruturas laríngeas^(28)^. Este método também permite caracterizar com maior riqueza de detalhes as irregularidades de vibração e duração do ciclo glótico, onda mucosa, relação vertical e padrão de fechamento glótico^(28)^.

A partir da análise do ciclo glótico de 252 participantes, de ambos os sexos, a HSV apresentou 100% de precisão diagnóstica nas alterações vocais de grau moderado e 64% das alterações de grau intenso quando comparada à videoendoscopia^(29)^. Pacientes com e sem alterações laríngeas foram avaliados pelos dois métodos e, os autores observaram diferenças significativas na classificação dos quadros vocais, uma vez que a HSV é capaz de fornecer imagens mais precisas da fonação ao longo de muitos ciclos glóticos, favorecendo a qualidade do diagnóstico^(30)^.

Os índices em saúde são desenvolvidos para permitir mensurar e atribuir valores que definem padrões, propiciando comparações e melhorando a precisão diagnóstica. A partir da observação dos parâmetros que melhor descrevem de forma concisa o evento, os índices auxiliam na compreensão e criação de valores de referência para a prática clínica, sendo utilizado de forma ampla em outras áreas do conhecimento^(31)^.

O considerável interesse na quantificação clínica da qualidade vocal, utilizando técnicas de análise acústica e análise perceptivo-auditiva, causou instigante desejo em pesquisadores para simplificação dessas medidas a índices numéricos. Na análise vocal existem índices descritos na literatura^(14-16,18)^, os quais consideram como material de análise a fala encadeada e vogais sustentadas.

Observou-se que, na avaliação da soprosidade vocal, proposta pelo *Acoustic Breathiness Breathiness Index* (ABI)^(18)^, os pesquisadores encontraram valores de sensibilidade e especificidade em 82,4% e 92,9%. Na avalição da qualidade vocal, a partir de medidas acústicas proposta pelo *Acoustic Voice Quality Index* (AVQI)^(16)^, foram encontrados valores de sensibilidade e especificidade de 85% e 100%, respectivamente, fornecendo parâmetros precisos para identificação e separação de vozes normais e disfônicas.

A triagem da gravidade da disfonia, a partir do CSID^(15)^, em 322 sujeitos, por meio do uso de medidas cepstrais, apresentou sensibilidade de 75% e especificidade de 73%, com possibilidade de acerto em 85%. E, a determinação do grau geral da disfonia, por meio do *Dysphonia Severity Index* (DSI)^(14)^ determinou valores de sensibilidade e especificidade de 72 e 75%, respectivamente.

O uso de recomendações sistemáticas (ou *guideliness)* para construção de práticas clínicas, a fim de sistematizar informações sobre determinadas condições de saúde, são amplamente difundidas^(17)^. Parâmetros como sensibilidade e especificidade são essenciais para determinar casos positivos e negativos de forma correta, os quais são aferidos a partir do teste padrão. Neste estudo, optou-se pelo uso da STARD para estabelecer critérios de construção, reduzindo possíveis vieses^(17)^.

A precisão diagnóstica de um teste clínico está atrelada aos seus valores de sensibilidade e especificidade. O FVI apresentou valor de corte de 0,71, sensibilidade de 76,4%, especificidade de 89,2%, com probabilidade de acerto de 81,5%. A determinação destes valores é primordial para estabelecer o poder diagnóstico de um instrumento. Os resultados do IVFG evidenciam altos valores de sensibilidade e especificidade, que determinam a capacidade preditiva deste teste diagnóstico^(15)^, confirmados pela área da Curva ROC de 89%, que caracteriza um poder de discriminação excelente^(22)^ entre mulheres com laringes normais e mulheres com laringe alterada em quadros de disfonia comportamental. Outros estudos^(16,18)^ também utilizaram a Curva ROC para determinar a especificidade e sensibilidade no diagnóstico por meio do índice, apresentando valores que variariam de 0,5 a 0,68. Os resultados obtidos por meio da curva ROC desta pesquisa reforçam o IVFG como um instrumento sensível e preciso na avaliação de laringes de mulheres com etiologia comportamental.

O IVFG, descrito nesse estudo, se torna um diferencial na qualidade do diagnóstico das alterações laríngeas de mulheres com disfonia comportamental, uma vez que utiliza uma tecnologia recentemente introduzida na avaliação laríngea.

São limitações desse estudo a aplicabilidade em indivíduos apenas do sexo feminino, que apresentem lesões laríngeas de caráter comportamental.

Todas as avaliações das imagens laríngeas e da qualidade vocal foram realizadas por consenso por profissionais especialistas na área com o objetivo de tornar a avaliação diagnóstica colaborativa. Outras possibilidades de avaliação diagnóstica são possíveis, como análise dos resultados mais prevalentes dos avaliadores com maiores níveis de concordância. Estudos futuros são necessários para uma melhor compreensão da melhor estratégia avaliativa e classificatória, quando se lida com análises subjetivas, como a análise perceptivo-visual da imagem laríngea, e a análise perceptivo-auditiva da voz.

A *American Speech Language-Hearing Association* (ASHA)^(2)^ recomendou protocolos para avaliação instrumental da produção de voz como a avaliação da imagem laríngea, a análise acústica da voz, e a avaliação aerodinâmica, com o objetivo de definir balizadores da avaliação vocal; permitir comparações entre diferentes centros clínicos e de pesquisa; e facilitar a avaliação da eficácia do tratamento vocal^(2)^. Segundo a ASHA, com relação à avaliação laríngea, recomenda-se a realização da videolaringoscopia para análise estrutural da laringe, e a videolarinestrobosgoscopia para avaliação do movimento vibratório das PPVV^(2)^. A literatura reforça que a imagem laríngea obtida pela videolaringoscopia é o padrão-ouro para o diagnóstico das alterações vocais^(32)^, e a HSV pode ser utilizada como um complemento para estroboscopia, principalmente para os quadros de disfonia caracterizadas por vibrações assimétricas ou aperiódicas das PPVV^(32)^. Apesar da HSV se configurar como uma tecnologia recente, dependente de um instrumental de alto custo, e ainda com pouco acesso à clínica laringológica, pesquisas científicas com a HSV são importantes para se analisar o real valor diagnóstico deste procedimento avaliativo.

Apesar da avaliação laríngea ser do escopo do médico otorrinolaringologista, a ASHA^(2)^ entende que o fonoaudiólogo deve ter conhecimentos e formação adequada para a análise funcional das imagens laríngeas, como auxílio na tomada da melhor decisão clínica para o tratamento fonoaudiológico de indivíduos disfônicos.

Pesquisas futuras com a aplicação do IVFG na determinação do diagnóstico de alterações laríngeas de mulheres com disfonia comportamental são importantes para se analisar a aplicação deste instrumento na prática clínica vocal.

## CONCLUSÃO

O IVFG apresenta valor de corte igual a 0,71, onde ≥0,71 representam presença de alterações laríngeas em quadros de disfonia comportamental, enquanto valores <0,71 determinam laringes normais. A probabilidade de acerto é de 81,5%, sensibilidade 76,4% e especificidade de 89,2%. O IVFG otimiza o diagnóstico laríngeo, e possibilita uma melhor definição do tratamento fonoterápico nos casos de disfonias comportamentais, potencializando o prognóstico na clínica vocal.
